# Efficacy and safety of *Ding-Kun-Dan* for female infertility patients with predicted poor ovarian response undergoing in vitro fertilization/intracytoplasmic sperm injection: study protocol for a randomized controlled trial

**DOI:** 10.1186/s13063-018-2511-0

**Published:** 2018-02-20

**Authors:** Saihua Ma, Ruihong Ma, Tian Xia, Masoud Afnan, Xueru Song, Fengqin Xu, Guimin Hao, Fangfang Zhu, Jingpei Han, Zhimei Zhao

**Affiliations:** 1 Reproductive Center, First Teaching Hospital of Tianjin University of Traditional Chinese Medicine, No. 88 Chang Ling Street, Xi Qing district, Tianjin, 300112 China; 2 Center of Reproductive Medicine, Tianjin United Family Hospital and Clinics, No. 22 Tanjiang Street, Hexi district, Tianjin, 300221 China; 3 Center of Reproductive Medicine, General Hospital of Tianjin Medical University, No. 154 Anshan Street, Nankai district, Tianjin, 300052 China; 4 Center of Reproductive Medicine, Tianjin First Center Hospital, No. 24 Fukang Road, Nankai district, Tianjin, 300190 China; 5 Center of Reproductive Medicine, The Second Hospital of Hebei Medical University, No. 215 Heping West Road, Xinhua district, Shijiazhuang, 050000 China; 6 The Graduate School, Tianjin University of Traditional Chinese Medicine, No. 312 Anshan West Road, Nankai district, Tianjin, 300073 China

**Keywords:** Traditional Chinese medicine, TCM, *Ding-Kun-Dan*, Poor ovarian reserve, Diminished ovarian reserve, Infertility, Assisted reproductive technology, IVF, ICSI

## Abstract

**Background:**

Women undergoing in vitro fertilization (IVF)/intracytoplasmic sperm injection (ICSI) who have a predicted poor ovarian response (POR) present a challenge for reproductive medicine specialists. Traditional Chinese medicine (TCM) is commonly used in China for such patients, in the belief that it will improve the ovarian response and ultimately increase pregnancy rates. However, there is a lack of high-quality evidence about the effect of TCM on improving ovarian response in such patients. The purpose of this study is to evaluate ongoing viable pregnancy rate at 12 weeks’ gestation and related indicators of ovarian response in fertile women who have a predicted poor ovarian response having immediate versus delayed IVF/ICSI after 3 months of *Ding-Kun-Dan* (DKD) pre-treatment.

**Methods/design:**

This study is a multicenter, randomized controlled, parallel-group, phase III, superiority clinical trial. Two hundred and seventy-eight eligible female infertility patients with POR will be included in the study and randomly allocated into an immediate treatment group and a DKD group in a 1:1 ratio. Both groups will receive IVF or ICSI as a standard treatment while in the DKD group, a commercially available Chinese medicine, DKD, will be administrated for 3 months before the IVF/ICSI cycle starts.

The primary outcome of the study is the ongoing pregnancy rate at 12 weeks’ gestation. The secondary outcomes include total gonadotropin dosage, duration of stimulation, estradiol (E_2_) and progesterone (P) levels on human chorionic gonadotropin (hCG) trigger day, cycle cancellation rate, number of oocytes retrieved, high-quality embryo rate, biochemical pregnancy rate, the change of serum anti-Müllerian hormone (AMH), follicle-stimulating hormone (FSH), and E_2_ levels and all side effects, safety outcomes, and any adverse events.

The protocol was approved by the Ethics Committee of the First Teaching Hospital of Tianjin university of TCM (approval no. TYLL2017[K] 004).

**Discussion:**

IVF/ICSI is increasingly used to treat couples desiring a baby. Many of these women will have poor ovarian function. In China, DKD is commonly used for these patients prior to undergoing IVF/ICSI. There is no effective treatment for poor ovarian response in Western medicine currently. It is important, therefore, to undertake this randomized control trial to determine whether DKD is effective or not.

**Trial registration:**

Chinese Clinical Trial Registry, ID: ChiCTR-IOR-17011697. Registered on 19 June 2017.

**Electronic supplementary material:**

The online version of this article (10.1186/s13063-018-2511-0) contains supplementary material, which is available to authorized users.

## Background

In order to achieve a pregnancy with in vitro fertilization (IVF)/intracytoplasmic sperm injection (ICSI), eggs must be retrieved from the woman. In order to increase the number of eggs, exogenous gonadotropins are given as part of controlled ovarian hyperstimulation (COH). The ideal ovarian response is to retrieve 10–15 eggs in order to maximize the chances of a successful pregnancy [1].

There is a worldwide trend to postpone childbearing due to socio-economic reasons, with the consequence that a number of women have a poor ovarian response (POR) prior to IVF, as the women are older. The low number of eggs collected dramatically lowers their chances of successful pregnancy in IVF/ICSI cycles [2], so much so that many IVF cycles are cancelled due to a POR [3].

Numerous treatment approaches, such as changing COH protocols [3–5], increasing gonadotropin dosage [6], using dehydroepiandrosterone (DHEA) [7–9] or other forms of androgen [10, 11], or adding growth hormone (GH) [12–14] have been tried in IVF/ICSI cycles to improve outcomes. While some show more promise than others, to date none have been shown to significantly improve pregnancy rates [15, 16].

Large numbers of reports indicate that traditional Chinese medicine (TCM) is useful for regulating hormone levels, improving implantation rates, and increasing pregnancy outcome in IVF cycles. Side effects and adverse reactions to TCM are much less frequent than with Western medicine [17]. Systematic reviews suggest that Chinese herbal medicine may be beneficial in increasing IVF/ICSI pregnancy rates [18–20]. However, the quality of the primary studies was considered poor [21].

*Ding-Kun-Dan* (DKD), as one of the most famous Chinese classic gynecological medicines, which was first used in the Qing Dynasty. It is commonly used in the treatment of irregular menstruation and infertility [22]. The components include red ginseng, cornu cervi, saffron, *Radix paeoniae alba*, *Radix rehmanniae*, *Angelica sinensis*, *Scutellaria baicalensis*, *Rhizoma cyperi*, *Rhizome chuanxiong*, motherwort fruit, *Rhizome corydalis*, among others. In TCM, DKD is used to improve renal and liver functions, nourishing the *qi* and blood, regulate menstrual function, dispel melancholy, promote blood circulation, and alleviate pain. It is useful for relieving symptoms of liver and kidney insufficiency, *qi*-blood deficiency and *qi*-blood stasis.

The purpose of this study is to evaluate ongoing viable pregnancy rate at 12 weeks’ gestation and related indicators of ovarian response in female fertile who have a predicted POR having immediate versus delayed IVF/ICSI after 3 months of DKD pre-treatment.

## Methods/design

### Study design

**Fig. 1:**
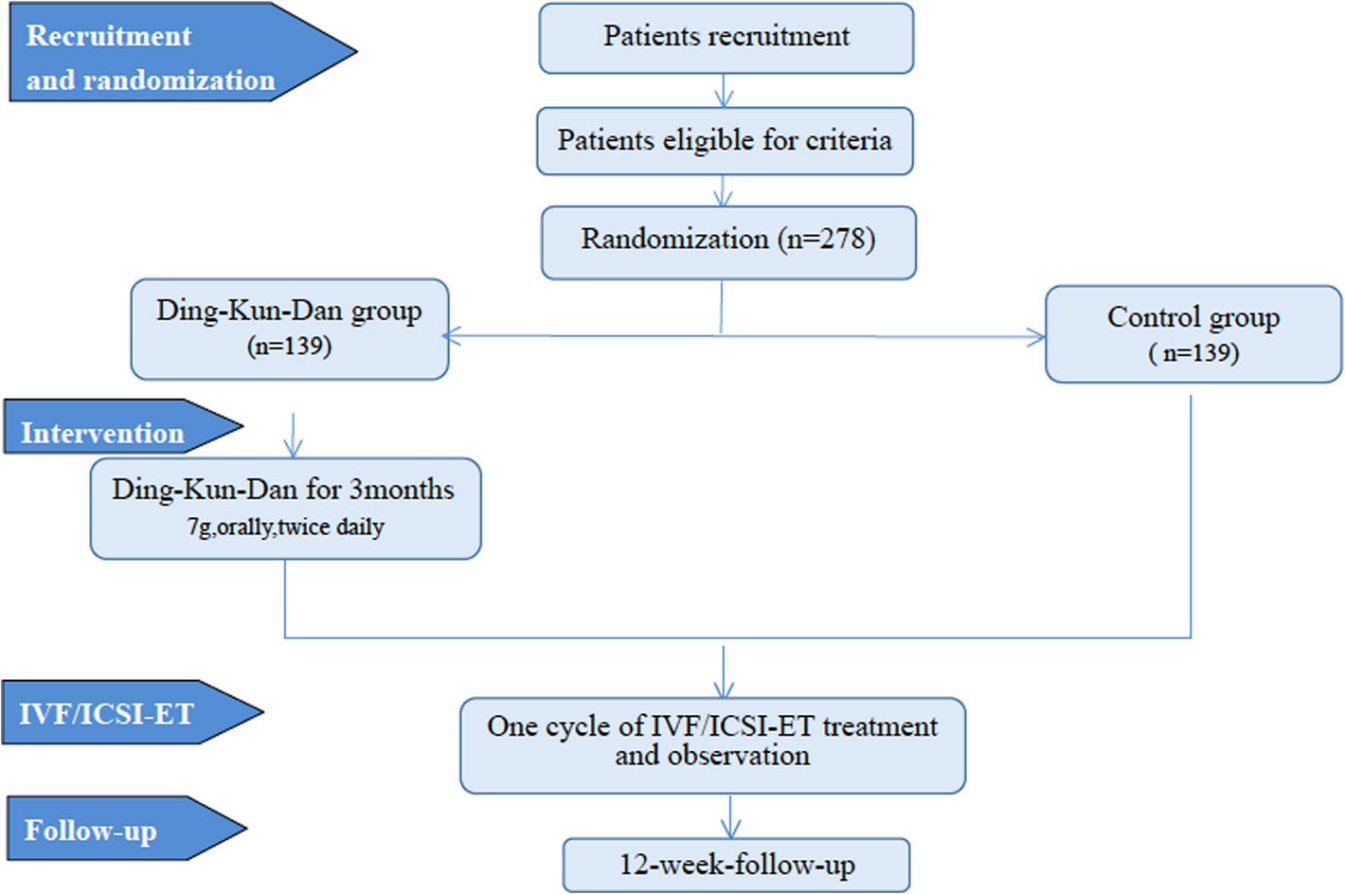
Study flow chart

This study is a multicenter, randomized controlled, parallel-group, phase III, superiority study. Eligible participants will be randomly assigned to the DKD group or the control group with a 1:1 ratio. The study flow is shown in Fig. 1. The study will be conducted in four stages in the DKD group and in three stages in the control group. Participants in the DKD group will receive DKD pre-treatment for 3 months before IVF/ICSI, while in the control group, participants will receive IVF/ICSI immediately without any other intervention.

The protocol complies with the principles of the Declaration of Helsinki as well as Good Clinical Practice (GCP) guidelines. The Standard Protocol Items: Recommendations for Interventional Trials (SPIRIT) Checklist can be found in Additional file 1.

### Participants

#### Recruitment

Potential participants will be recruited from Tianjin Medical University General Hospital (Tianjin, China), Tianjin First Center Hospital (Tianjin, China) and The Second Hospital of Hebei Medical University (Hebei, China). The participating center must have an IVF program and be able to participate fully in the study. In China, IVF centers are regulated and typically located in level-3 (the highest level) hospitals.

**Fig. 2:**
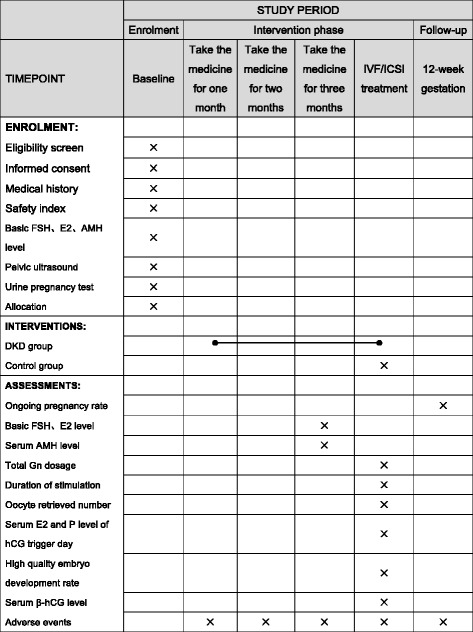
Standard Protocol Items: Recommendations for Interventional Trials (SPIRIT) Figure

Our study will be propagated via the Internet, bulletin boards, and posters in communities and hospitals, with a hotline provided for potential volunteers to call. Potential participants will be asked and talk face to face with researchers, and will be given detailed information about the study. If patients are eligible and interested in participating, they will be asked to sign an informed consent. An overview of specific measurements and time points of data collection can be found in the SPIRIT Figure (Fig. 2).

#### Randomization and allocation

Eligible participants will be randomly assigned to the experimental group (DKD for 3 months, then IVF/ICSI) or the control group (immediate IVF/ICSI) in a 1:1 ratio by central randomization performed by an independent statistician from Tianjin CLINDA Medical Technology Co., Ltd. Random numbers will be generated by using dynamic randomization on an online computer, which will generate a randomization schedule. Recruitment, allocation, instructions how to take DKD, and study investigations will be carried out by the study investigators. The IVF/ICSI is carried out by the treating team.

#### Sample size

The sample size calculation is based on the ongoing pregnancy rate. Studies indicated that ongoing pregnancy rate of female infertility with POR undergoing IVF/ICSI cycles was 12.5% to 15.8%, with an average of 13% [23–27]. We hypothesized that a supplementation of DKD can increase the ongoing pregnancy rate to 26%.

According to the sample size of the estimation formula [28]:

$$ n\kern0.5em =\kern0.5em {\left({Z}_{\alpha }+\kern0.5em {Z}_{\beta}\right)}^2\kern0.5em \left[{P}_1\left(1-{P}_1\right)+{P}_2\left(1-{P}_2\right)\right]/{\left(\varepsilon -\delta \right)}^2, $$

where *n*_1_ = *n*_2_ = *n*/(1–20%), *Z*_α_ = Z_0.05_ = 1.64, *Z*_β_ = *Z*_0.8_ = 0.84, *P*_1_is the ongoing pregnancy rate of the treatment group = 0.26, *P*_2_ is the ongoing pregnancy rate of the control group = 0.13, and *ε* is the difference of *P*_1_ and *P*_2_. Assume the superiority margin is 0 (*δ* = 0).

For 80% power to detect a difference between the experimental arm and the control arm at a one-sided 5% type I error rate, we need 112 per arm. This assumes standard sample size formulae for binary outcomes using a normal approximation [28]. To account for a 20% dropout rate, we will recruit 139 patients per arm. Considering the number of outpatients in each centre will be fluctuating during the trial; therefore, we use a dynamic randomization method to generate random numbers, and so there is no strict restriction on the number of participants in each centre.

#### Inclusion criteria

Patients are eligible to be included if they meet the following criteria: (1) are female, aged between 25 and 38 years, (2) have regular menstrual cycles of between 25 to 35 days, (3) are diagnosed as likely to have a POR where at least two of the following three features must be present: at least five oocytes in a previous IVF/ICSI treatment cycle with a conventional stimulation protocol; combined antral follicle count of less than seven; or anti-Müllerian hormone (AMH) < 1.1 ng/ml, (4) diagnosed with infertility (aged under 35 years, the duration of infertility is more than 12 months and, for those who over 35 years, their duration of infertility can be shortened to 6 months or any other factors which are likely to cause infertility), (5) planning to do IVF/ICSI, and (6) competent and able to give informed consent.

#### Exclusion criteria

Patients who present with any of the following conditions will be excluded: (1) other endocrine disease or autoimmune disease, e.g., polycystic ovarian syndrome (PCOS), hyperprolactinemia, thyroid dysfunction, diabetes etc., (2) any other factors known to reduce pregnancy rates in IVF/ICSI cycles, e.g., endometriosis, intrauterine adhesions, sub-mucosal fibroids, significant uterine malformations, hydrosalpinx, etc., (3) suffering serious medical, surgical or mental illness, (4) a hereditary disease or other serious illness which would be a contraindication to pregnancy, (5) allergy to any ingredient in DKD, (6) use of hormones or TCM (contraceptive drugs, ovulation-stimulating medicine, androgens, and glucocorticoid, etc.) within 3 months prior to study entry.

#### Discontinuation criteria

Reasons for discontinuation of treatment may include, but are not limited to, the following: (1) participants become pregnant during the trial, (2) participants who have experienced some complications or serious side effects), (3) participants who do not comply with the DKD regimen, as defined by less than 80% or more than 120% of the prescribed amount; using prohibited drugs as described in the protocol, such as contraceptive drugs, ovulation-stimulating medicine, glucocorticoids etc., (4) participants who request to withdraw from the trial.

### Intervention

#### DKD group

The study will be conducted in four stages in the DKD group. After a recruitment period prior to baseline assessment and randomization, DKD (water-honeyed pill) will be given to patients in the experimental group 7 g twice daily orally from the first day of their menstrual period, for 3 months. If the participant has side effects on this dose, it is permissible to reduce the dose to 3.5 g twice daily. After that, participants will undergo their IVF/ICSI cycle. Finally, a 12-week follow-up will be included.

COH prior to IVF/ICSI will be initiated on day 3 of the menstrual cycle. All participants will receive a fixed initial daily follicle-stimulating hormone (FSH) (Gonal F, Merck Serono SA, Switzerland) dose of 300 IU subcutaneously for the first 5 days [29]. Thereafter, the daily dose will be adjusted up or down according to follicular response and serum estradiol (E_2_) level, with 450 IU as the maximum daily dose allowed. Participants will receive a gonadotropin-releasing hormone (GnRH) antagonist (Cetrorelix Ac, Merck Serono, Darmstadt, Germany, or ORGALUTRAN, Merck, Kenilworth, NJ, USA) when the dominant follicle reaches 14 mm in diameter or serum E_2_ level higher than 350 pg/ml, at a dose of 0.25 mg daily subcutaneously throughout the remainder of the stimulation period. Triggering of final follicular maturation is performed as soon as one or more follicles are ≥ 18 mm in diameter with 250 μg recombinant hCG (choriogonadotropin-α, Ovitrelle, EMD Serono, Darmstadt, Germany).

Transvaginal ultrasound-guided oocyte retrieval will take place 36 ± 2 h after triggering of final follicular maturation. Oocytes will be inseminated using conventional IVF or ICSI, according to the clinical indication. Embryo transfer will be performed on days 2–3 after oocyte retrieval and a maximum of two embryos will be transferred. Surplus embryos will be cultured until the blastocyst stage and subsequently cryopreserved, for use after trial completion.

Vaginal progesterone gel (Crinone gel, 8%, Merck Serono, Switzerland) 90 mg once daily or dydrogesterone tablet (Duphaston, Abbott, Netherlands) 10 mg twice daily orally will be used for luteal-phase support from the day after oocyte retrieval for 13–15 days. If serum hCG positive, progesterone is continued until 10–12 weeks’ gestation. If hCG is negative, or if the pregnancy fails, P support is stopped. Ultrasound will be performed at 4–6 weeks and 10–12 weeks after embryo transfer to confirm clinical and ongoing viable pregnancy, respectively.

#### The control group

The study will be conducted in three stages in the control group. After a recruitment period prior to baseline assessment and randomization, patients in the control group will be arranged to accept IVF/ICSI directly without any other intervention. The participants in this group will receive the GnRH antagonist protocol for COH that is the same as the DKD group. Finally, a 12-week follow-up will be included.

#### General advice

All participants in both groups will be advised to maintain a healthy lifestyle during the study, such as no smoking, no alcohol, low-fat high-protein diets, regular exercise, and to get plenty of sleep.

#### Management of intervention medication (DKD)

All the DKD (water-honeyed pill) will be provided free of charge by the Shanxi Guangyuyuan TCM Co., Ltd. It will be stored at room temperature and dispensed locally at the treating hospital. Patients will take DKD 7 mg twice daily orally from the first day of their menstrual period for 3 months. If the patient has side effects on this dose, it is permissible to reduce the dose to 3.5 mg twice daily. If on completion of the trial, or in case of withdrawal from the study, any unused DKD will be returned to the pharmacy. As DKD is the standard of care within TCM, no special drug accountability is required. Patients will be given a diary and asked to keep a record of their daily medication usage, which is returned to the investigator on completion of the course of DKD treatment. A complete listing of all concomitant medication received during the treatment phase must be recorded in the relevant Case Report Form (CRF).

#### Patient safety and indemnity

DKD is the standard of care within TCM. The TCM hospital in which the DKD is prescribed, and the hospital in which the IVF/ICSI treatment is carried out, provide indemnity for approved clinical research in their institution.

As DKD is the standard of care, no special arrangements need to be put in place in case of adverse events.

In case of emergency, the patient will be provided with a 24-h contact number to call.

### Outcome measures

#### Primary outcome

The primary outcome of the study is ongoing pregnancy rate, defined by at least one intrauterine viable fetal heart beat at 12 weeks of gestation.

#### Secondary outcomes

The secondary outcomes include total gonadotropin dosage; duration of stimulation; cycle cancellation rate; E_2_ and progesterone (P) levels on the hCG trigger day; retrieved oocyte number; high-quality embryo development rate; biochemical pregnancy; as well as some specific endpoints indicative of the ovarian response, such as the change of serum AMH, FSH, E_2_ level. All side effects, safety outcomes and adverse events will also be recorded. All data will be collected using an electronic Case Report Form (eCRF) and will be supervised by an independent statistician from Tianjin CLINDA Medical Technology Co., Ltd.

The standard of high-quality embryo development rate is according to the Istanbul criteria [30] as following: Grade 1 – good rating: 10% fragmentation, stage-specific cell size, no multinucleation. Grade 2 – fair rating: 10–25% fragmentation, stage-specific cell size for majority of cells, no evidence of multinucleation. Grade 3 – poor rating: severe fragmentation (> 25%), cell size not stage specific, evidence of multinucleation.

#### Adverse events

During the study, all the details including all adverse events, such as swelling and aching of the gums, oral ulceration, constipation, and ovarian hyperstimulation (OHSS) will be recorded in detail. Serious adverse events will be immediately reported to the principal investigator, and appropriate measures will be initiated instantly.

The Ethics Committee will determine whether the adverse event is likely to have been associated with the experimental drug, and the sponsor will provide the cost of the treatment associated with the study and the corresponding financial compensation.

#### Data management

All data will be collected using an eCRF and will be supervised by an independent statistician from Tianjin CLINDA Medical Technology Co., Ltd., and will be completed and recorded on the paper and eCRF. Paper files will be kept in a locked filing cabinet in the treating hospital. Electronic documents will be stored in a password-protected computer, with access restricted to the principal investigator. All research documents will be preserved for at least 5 years after publication.

#### Quality control

All clinicians involved with the trial will be fully trained in the trial methodology. After the clinician has recruited the first patient the CRF will be reviewed by the researcher to ensure that it has been correctly completed.

To ensure trial quality and reliability, a clinical trial monitor from Tianjin Taicheng Yaozhong Biomedical Technology Co., Ltd. will verify all of the process details at when the experiment is half done and completed. Moreover, the monitor will check the authenticity, accuracy, and integrity of the data.

#### Collecting trial data

At the beginning of the study, blood will be taken for baseline FSH, E_2,_ and AMH, and an ultrasound will be performed to measure the antral follicle (AFC) in both groups. After 3 months of DKD treatment, and before commencing IVF/ICSI, the same data will be collected in the DKD group. These blood tests will be assayed within each participating center. The AFC will be performed by the treating team.

After the IVF/ICSI treatment cycle has been completed, the total gonadotropin dosage, duration of stimulation, E_2_ and P  levels on hCG trigger day, cycle cancellation rate, retrieved oocyte number, high-quality embryo rate, and biochemical pregnancy will be recorded.

If the patient is pregnant, the presence or absence of a fetal heart will be noted 4–6 weeks after the embryo transfer, and the ongoing pregnancy rate at 12 weeks of gestation, and the miscarriage rate will be recorded during the follow-up.

Trained nurses will take the blood samples, and ultrasound physicians, independent of the researchers, will perform the ultrasonography.

### Statistical analysis

Statistical analysis will be performed using SAS 9.2 software package. Intention-to-treat analysis will be applied to minimize bias due to dropouts, and reasons for which patients have dropped out will be recorded in detail and analyzed after the trial. Multiple imputation analysis will be used for missing data. Normally distributed continuous variables are reported as means and standard deviations, non-normally distributed continuous variables are reported as medians and ranges, and the count data will be described by frequency, percentage and constituent ratio. Results will be presented as point estimates and 95% confidence intervals; the level of significance will be set at 5%.

Baseline characteristics of dropout patients will be compared with those who did not. As age is a crucial factor for infertility and the effect of treatment, a simple linear model with age as a covariate will be used to analyze continuous outcomes, and a binomial generalized linear model (GLM) with age as a covariate for binary outcomes.

To compare the efficacy of DKD for female infertility with POR with the control group, the Pearson’s chi-squared test will be applied to compare frequencies between groups such as the ongoing pregnancy rate, cycle cancellation rate, high-quality embryo rate. And the *t* test will be performed to compare continuous parameters (retrieved oocyte number, gonadotropin dosage, E_2_ and P level on the hCG trigger day, etc.) between the groups. In the treatment group, the rank sum test or paired-sample *t* tests will be used to compare the indicators pre- and post treatment; for instance, basic FSH, E_2_ level, and AMH level.

#### Ethical considerations

The trial protocol is in accordance with the principles of the Declaration of Helsinki and was approved by the Ethics Committee of the First Teaching Hospital of Tianjin University of TCM (approval no. TYLL2017[K] 004). This trial was registered at the Chinese Clinical Trail Registry, (ID: ChiCTR-IOR-17011697). Each participant will be fully informed regarding the study protocol. Written informed consent will be obtained from every participant.

## Discussion

POR has been a great challenge in the field of reproductive medicine. At present, there is no single intervention generally accepted as beneficial in improving ovarian reserve greatly. A review indicated that only 10% of randomized controlled trials (RCTs) reported significant improvement in reproductive outcomes among tested interventions [2]. Therefore, other new treatment options are urgently needed to improve the poor IVF outcomes in this patient population.

On the basis of more than 3000 years of clinical practice, TCM has been making an indelible contribution to human health. In 2015, Chinese pharmacologist Youyou Tu was awarded the Nobel prize in physiology or medicine for the discovery of the new antimalarial drug artemisinin and dihydroartemisinin, which was a milestone on the road of TCM development. This inspiring event encouraged the TCM community to increasingly share the benefits of TCM with Western medicine. In order to achieve this aim, TCM will need to integrate progressively, using the concepts and methods of evidence-based medicine. Research in TCM need to be transformed and improved. DKD has been widely used in the treatment of infertility, but useful empirical research is insufficient for its popularization and application. A large proportion of TCM studies to date are not good enough to convince people of its value due to less rigorous designs, small sample size and low quality.

This study is a pragmatic RCT to assess the efficacy of DKD in infertile women with POR. The main limitation of the study is that it is not blinded and is not placebo controlled. A survey has shown that 96% of POR patients would refuse to participate in a clinical trial that might include a placebo group [9]. As patients with POR have declining (over time) fertility [31], they are unwilling to “waste” 3 months to receive placebo once they are diagnosed as having POR. Therefore, eligible participants in the control group of our study will be allocated to enter the IVF cycle without any delay.

In this study, the participants’ age will be limited to 25 to 38 years in the study as age is recognized as an independent adverse factor for ovarian response, and this tendency is usually not reversible by any drug. González-Foruria and his team analyzed 947 natural IVF cycles and found that both the ongoing pregnancy rate (3%) and the miscarriage rates (50%) were significantly worse in the older groups (40 years and older). Age is an independent variable factor which has a great impact on ongoing pregnancy rate and miscarriage rate [32]. A similar conclusion was also obtained among the Chinese POR patients by Zhen et al. [33].

Another limitation in this study is the primary outcome. Couples desire a live healthy baby, and there is a recognized miscarriage and fetal loss rate after 12 weeks, though this is < 5%. In 2003, ESHRE recommended the singleton live birth rate as the evaluation index of assistedreproductivetechnology(ART) or non-ART reproductive outcome [34], but the proposal has not been widely applied in clinical research: the proportion of trials that used the singleton live birth rate as primary outcome was just 7% before and 9% after the recommendation [35]. The reason may be due to the difficulties caused by prolonging the trial, thereby adding to the cost and the increased risk of dropout. Furthermore, it is thought unlikely that obstetric factors which might result in a pregnancy loss would be influenced by the DKD. For these reasons, we chose the ongoing pregnancy rate as the primary outcome measure.

For POR patients treated with IVF, the most common ovarian stimulation protocols are for GnRH antagonists and GnRH agonists. However, studies have shown that there is no significant difference in the number of retrieved oocytes, transfer rate, cycle cancellation rate, ongoing pregnancy rate and clinical pregnancy rate between the two protocols for POR patients [15, 36, 37]. GnRH antagonists compete with GnRH receptors in the anterior pituitary gland, inhibiting gonadotropin release immediately, preventing premature release of endogenous LH and FSH. A GnRH antagonist has the advantages of quick onset and short action time, the inhibition is dose-dependent, and the pituitary function recovers rapidly after the withdrawal. So, in this study, we use the GnRH antagonist protocol in IVF/ICSI cycle.

### Trial status

This trial was initiated in May 2017 and has currently recruited 40 participants in three centers.

## Additional file

Additional file 1: SPIRIT Checklist. The SPIRIT Checklist document. (DOC 135 kb)

## Abbreviations

AFC: Antral follicle
AMH: Anti-Müllerian hormone
ART: Assisted reproductive technology
COH: Controlled ovarian hyperstimulation
CRF: Case Report Form
E_2_: Estradiol
FSH: Follicle-stimulating hormone
GnRH: Gonadotropin-releasing hormone
hCG: Human chorionic gonadotropin
ICSI: Intracytoplasmic sperm injection
IVF: In vitro fertilization
P: progesterone
PCOS: Polycystic ovarian syndrome
POR: Poor ovarian response
RCT: Randomized controlled trial
TCM: Traditional Chinese medicine
