# Supplementary material for: Efficacy and safety of Ding-Kun-Dan for female infertility patients with predicted poor ovarian response undergoing in vitro fertilization/intracytoplasmic sperm injection: study protocol for a randomized controlled trial
Source: Trials. 2018 Feb 20;19:124. doi: 10.1186/s13063-018-2511-0 (PMC5819272; doi:10.1186/s13063-018-2511-0)
Supplement: Supplementary file 1 — SPIRIT Checklist. The SPIRIT Checklist document. (DOC 135 kb) [file 13063_2018_2511_MOESM1_ESM.doc]

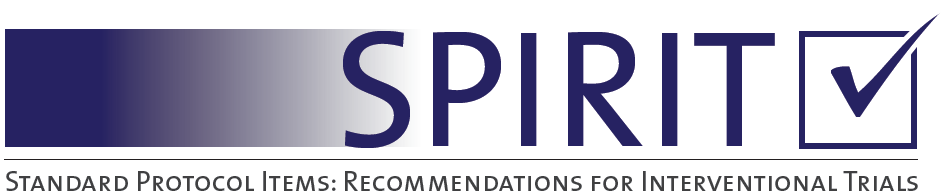


SPIRIT 2013 Checklist: Recommended items to address in a clinical trial protocol and related documents*

| Section/item | Item No | Description | | | Addressed on page number |
| --- | --- | --- | --- | --- | --- |
| **Administrative information** | | | |  |  |
| Title | 1 | | Efficacy of traditional Chinese medicine (Ding-Kun-Dan) for female infertility with poor ovarian response (POR): study protocol for a randomized controlled trial | | 1 |
| Trial registration | 2a | | Trial Registration Number: ChiCTR-IOR-17011697. | | 2 |
|  | 2b | |  | |  |
| Protocol version | 3 | | 2016-12-15 Version 1.0 | |  |
| Funding | 4 | | The study was supported by a generous grant from Shanxi Guangyuyuan TCM Co., Ltd. The funders had no role in study design, data collection and analysis, decision to publish, or preparation of the manuscript. | | 19 |
| Roles and responsibilities | 5a | | TX and MA designed the whole study. SM and RM conducted data analyses and drafted the manuscript. FZ and JH participated in the manuscript writing and contributed to this protocol. TX and MA read and approved the final manuscript. XR, FX and GH supervised patient diagnosis and recruitment. All authors critically reviewed the article and approved the final manuscript. | | 19-20 |
|  | 5b | | Reproductive Center, First Teaching Hospital of Tianjin University of Traditional Chinese Medicine (Telephone number: 0086 022 27987330) | |  |
|  | 5c | | Shanxi Guangyuyuan TCM Co., Ltd. is the funders, and had no role in study design, data collection and analysis, decision to publish, or preparation of the manuscript. | |  |
|  | 5d | | The trial will be conducted at Tianjin Medical University General Hospital, Tianjin First Center Hospital and The Second Hospital of Hebei Medical University. Tianjin CLINDA Medical Technology Co.,Ltd. is in charge of data management. | |  |
| Introduction |  | |  | |  |
| Background and rationale | 6a | | Large numbers of reports indicate that traditional Chinese medicine (TCM) is useful for regulating hormone levels, improving implantation rate and increasing pregnancy outcome in IVF cycles. Side effects and adverse reactions to TCM are much less than with western medicine. Systematic reviews indicated that Chinese herbal medicine is beneficial to increase IVF successful rate. However, there are many defects in these trials, for example, without a strict standard clinical scheme design, irrational grouping and inappropriate drug administration. Thus, the results of these studies are still controversial. Here we present a rigorously designed study for assessing the efficacy of DKD in the treatment of patients with POR. | | 3-4 |
|  | 6b | | In the control group, patients will be arranged to accept IVF/ICSI directly. A survey showed that 96% of POR patients would refuse to participate in a clinical trial which might include a placebo group. Patients understand that their reproductive potential is declining, and they are not willing to ‘waste’ 3 months once they are diagnosed as having POR. Therefore, eligible participants in the control group of our study will be allocated to enter IVF cycle without any delay. | | 5, 16-17 |
| Objectives | 7 | | This study aims to evaluate the efficacy and of DKD treatment in women undergoing IVF/ICSI with POR. More importantly, it will be a new attempt of verifying the value of TCM in patients with POR in assisted reproductive technology. | |  |
| Trial design | 8 | | A multicenter randomized controlled trial | | 4 |
| Methods: Participants, interventions, and outcomes | | | |  |  |
| Study setting | 9 | A multicenter randomized controlled trial will be conducted at the First Teaching Hospital of Tianjin University of TCM, Tianjin Medical University General Hospital, Tianjin First Center Hospital and The Second Hospital of Hebei Medical University. | | | 4 |
| Eligibility criteria | 10 | Patients are eligible to be included if they meet the following criteria: (1) Female, aged between 25 and 38 years; (2) have regular menstrual cycles between 25 to 35 days; (3) diagnosed as likely to have a poor ovarian response (POR), at least two of the following three features must be present: ≤5 oocytes in a previous IVF/ICSI treatment cycle with a conventional stimulation protocol; combined antral follicle count of < 7; or anti-mullerian hormone (AMH) < 1.1 ng/ml (4) diagnosed with infertility (aged under 35 years, the duration of infertility is more than 12 months and for those who over 35 years, their duration of infertility can be shortened to 6 months or any other factors which are likely to cause infertility); (5) planning to do IVF/ICSI; (6) competent and able to give informed consent.  Patients who present any of the following criteria will be excluded: (1) other endocrine disease or autoimmune disease, e.g. polycystic ovarian syndrome (PCOS), hyperprolactinemia, thyroid dysfunction, diabetes etc; (2) any other factors known to reduce pregnancy rates in IVF/ICSI cycles, e.g. endometriosis, intrauterine adhesions, sub-mucous fibroids, significant uterine malformations, hydrosalpinx, etc; (3) suffering serious medical, surgical or mental illness; (4) a hereditary disease or other serious illness which would be a contraindication to pregnancy; (5) allergy to any ingredient in DKD; (6) use of hormones or TCM (contraceptive drugs, ovulation-stimulating medicine, glucocorticoid etc) within 3 months prior to study entry. | | | 6-7 |
| Interventions | 11a | Ding-Kun-Dan (water-honeyed pill) will be given to patients 7g twice daily orally from the first day of their menstrual period, for 3 months. If the participant has side-effects on this dose, it is permissible to reduce the dose to 3.5g twice daily. After that, participants will undergo their IVF/ICSI cycle. In the control group, patients will be arranged to accept IVF/ICSI directly without any other intervention. Everyone in each group will receive gonadotropin releasing hormone antagonist protocol for controlled ovarian hyperstimulation (COH). | | | 9-10 |
|  | 11b | Reasons for discontinuation of treatment may include, but are not limited to, the following: (1) Participants get pregnant during the trial; (2) Participants who had some complications or serious side effects); (3) Participants who do not comply with the DKD regimen, as defined by less than 80% or more than 120% of the prescribed amount; Using prohibited drugs as described in the protocol, such as contraceptive drugs, ovulation-stimulating medicine, glucocorticoid etc.; (4) Participants who request to withdraw from the trial. | | | 8 |
|  | 11c | Patients will be given a diary and asked to keep a record of their daily medication usage, which is returned to the investigator on completion of the course of DKD treatment. | | | 10 |
|  | 11d | The patient will not be allowed to take hormonal therapy or concomitant TCM which might interfere with the study. | | | 10 |
| Outcomes | 12 | The primary outcome of the study is ongoing pregnancy rate. The secondary outcomes include The secondary outcomes include total gonadotropins dosage; duration of stimulation; cycle cancellation rate; E2 and P level of hCG trigger day; retrieved oocyte number; high quality embryo development rate; biochemical pregnancy; as well as some specific endpoints indicative of the ovarian response, such as the change of serum AMH, FSH, E2 level. Besides, safety outcomes and adverse events will be also necessary. | | | 11-12 |
| Participant timeline | 13 | Figure 1 | | |  |
| Sample size | 14 | The sample size calculation is based on the ongoing pregnancy rate. Studies indicated that ongoing pregnancy rate of infertility women with POR undergoing IVF/ICSI cycles was 12.5% to 15.8%, with an average of 13%. We hypothesized that a supplementation of DKD can increase the ongoing pregnancy rate to 26%. According to the sample size of the estimation formula: n_1_=n_2_=(Z_α_+Z_β_)^2^[P_1_(1-P_1_)+P_2_(1-P_2_)]/δ^2^, Z_α_= Z_0.05_=1.64，Z_β_= Z_0.8_=0.84, P_1_ is the ongoing pregnancy rate of treatment group = 0.26, P_2_ is the ongoing pregnancy rate of the control group = 0.13, δ is the difference of P_1_ and P_2_. It is estimated that a sample size of 139 participants per group will be required, considering a 25% dropout. | | | 6 |
| Recruitment | 15 | Participants will be recruited for Tianjin Medical University General Hospital(Tianjin, China), Tianjin First Center Hospital(Tianjin, China) and The Second Hospital of Hebei Medical University(Hebei, China). | | | 5 |
| **Methods: Assignment of interventions (for controlled trials)** | | | |  |  |
| Allocation: |  |  | | |  |
| Sequence generation | 16a | The participants who meet the inclusion criteria will be than randomly assigned to the experimental group or the control group in a 1:1 ratio by central randomization performed by an independent statistician from Tianjin CLINDA Medical Technology Co.,Ltd. Random numbers will be generated by using dynamic randomization, an online computer generated randomization schedule. | | | 5 |
| Allocation concealment mechanism | 16b | - | | |  |
| Implementation | 16c | - | | |  |
| Blinding (masking) | 17a | The main limitation of the study is that it is not blinded and is not placebo controlled. A survey showed that 96% of POR patients would refuse to participate in a clinical trial which might include a placebo group. Patients understand that their reproductive potential is declining, and they are not willing to ‘waste’ 3 months once they are diagnosed as having POR. Therefore, eligible participants in the control group of our study will be allocated to enter IVF cycle without any delay. Which will encourage patient recruitment. | | | 16-17 |
|  | 17b | - | | |  |
| **Methods: Data collection, management, and analysis** | | | |  |  |
| Data collection methods | 18a | At the beginning of the study, blood will be taken for baseline FSH, E2 and AMH, and an ultrasound will be performed to measure the AFC in both groups. After 3 months of DKD treatment, and before commencing IVF/ICSI, the same data will be collected in the DKD group. These blood tests will be assayed within each participating Center. The AFC will be performed by the treating team.  After the IVF/ICSI treatment cycle has been completed, the total gonadotropins dosage, duration of stimulation, E2 and P level of hCG administration, cycle cancellation rate, retrieved oocyte number, high quality embryo rate, and biochemical pregnancy will be recorded.  If the patient is pregnant, the presence or absence of a fetal heart will be noted 4-6 weeks after the embryo transfer, and the ongoing pregnancy rate at 12 weeks of gestation, and the miscarriage rate will be recorded during the follow-up.  Trained nurses will take the blood samples, and ultrasound physicians, independent of the researchers, will perform the ultrasonography. | | | 13-14 |
|  | 18b | Dropouts and withdrawals from the study will be recorded in detail | | |  |
| Data management | 19 | All data will be collected using an electronic case report form and will be supervised by an independent statistician from Tianjin CLINDA Medical Technology Co.,Ltd., and will be completed and recorded on the paper and electronic case report form (CRF). Paper files will be kept in a locked filing cabinet in the treating hospital. Electronic documents will be stored in a password protected computer, with access restricted to the principal investigator. All research documents will be preserved for at least 5 years after publication. | | | 13 |
| Statistical methods | 20a | To compare the efficacy of DKD for female infertility with POR with the control group, the chi-squared test or Wilcoxon rank sum test will be applied to compare frequencies between groups such as the ongoing pregnancy rate, cycle cancellation rate, high quality embryo rate. And rank sum test or t-test will be performed to compare continuous parameters (retrieved oocyte number, gonadotropin dosage, E2 and P level on hCG trigger day, etc) between the groups. In the treatment group, rank sum test or paired-sample t-tests will be used to compare the indicators pre-and post-treatment, for instance, basic FSH, E2 level, and AMH level. | | | 15 |
|  | 20b | - | | |  |
|  | 20c | Intention-to-treat analysis will be applied to minimise bias due to dropouts, and reasons for which patients have dropped out will be recorded in detail and analyzed after the trial. | | | 14 |
| **Methods: Monitoring** | | | |  |  |
| Data monitoring | 21a | This trial will be monitored by Tianjin TICE biological medicine technology Co., Ltd. | | |  |
|  | 21b | - | | |  |
| Harms | 22 | During the study, all of the details including all of adverse events such as swelling and aching of gum, oral ulcer, constipation, ovarian hyperstimulation (OHSS) will be recorded in detail. Serious adverse events will be immediately reported to the principal investigator, and appropriate measures will be initiated instantly. | | | 12 |
| Auditing | 23 | The study will be audited by Tianjin TICE biological medicine technology Co., Ltd. every year. | | |  |
| Ethics and dissemination | | | |  |  |
| Research ethics approval | 24 | The protocol was approved on May 9, 2017, by the Ethics committee of the First Teaching Hospital of Tianjin university of TCM (approval no. TYLL2017[K] 004). | | | 15 |
| Protocol amendments | 25 | If it is necessary to modify the protocol, we should submit applications to the Ethics committee of the First Teaching Hospital of Tianjin university of TCM | | |  |
| Consent or assent | 26a | Researchers from three centers will obtain informed consent or assent from potential trial participants. Participants will sign informed consent. | | |  |
|  | 26b | - | | |  |
| Confidentiality | 27 | Research data will be gathered and saved. Paper files will be kept in a locked filing cabinet. Electronic documents will be stored in a password protected computer, with access restricted to the principal investigator. All research documents will be preserved for at least 5 years after publication. | | | 13 |
| Declaration of interests | 28 | No competing interests | | |  |
| Access to data | 29 | Dataset will be stored in a computer which will be password protected, with access restricted to the principal investigator | | | 13 |
| Ancillary and post-trial care | 30 | - | | |  |
| Dissemination policy | 31a | The results will be published after the study. | | |  |
|  | 31b | Researchers in this trial will have authorship eligibility. | | |  |
|  | 31c | The results will be published. | | |  |
| Appendices |  |  | | |  |
| Informed consent materials | 32 | Written informed consent will be obtained from all participants. | | |  |
| Biological specimens | 33 | Serological indicators will be detected by unified Kits. | | |  |

*It is strongly recommended that this checklist be read in conjunction with the SPIRIT 2013 Explanation & Elaboration for important clarification on the items. Amendments to the protocol should be tracked and dated. The SPIRIT checklist is copyrighted by the SPIRIT Group under the Creative Commons “[Attribution-NonCommercial-NoDerivs 3.0 Unported](http://www.creativecommons.org/licenses/by-nc-nd/3.0/)” license.
